# Mr. Dunning, in Reply to Dr. Walker

**Published:** 1805-06-01

**Authors:** Richard Dunning

**Affiliations:** Plymouth Dock


### *To the Editors of the Medical and Physical Journal.*

GENTLEMEN,

I Rejoice that Dr. Walker has noticed my late Letter in the Star, without indulging so largely in those veins of flippancy and asperity, which have for some time past so much characterized his papers on the benign subject of Vaccination; and I rejoice too, because I think I read in his remarks, a confirmation and demonstration of the opinions I have undertaken to suggest. Nothing, I think, can be more obvious to every person who does not read and reason very superficially, or reason at all indeed, that the Doctor goes far apparently to subscribe opinions which he professes to combat, and to give to airy nothings or notions, as he terms them, a local habitation and a name. The indefatigable secretary, and vaccinator, (no man, I really believe, is more justly entitled to this distinction) at once tells us, "*That from the constant demands for matter at the Central House, he is daily obliged to break down pocks at all stages from their first formation till they have acquired their full dimensions, and then adds, that the parents are assured* that their children are perfectly protected.*" Now, gentlemen, at Plymouth, we had anticipated this information, for we understood several cases of secondary variolæ at least had occurred in subjects who had been vaccinated at Head Quarters; and we now venture to predict that more will occur. Without further preface, therefore, (Dr. Walker is not a man to stand upon compliments, nor do present circumstances seem to require them) we beg leave to call earnestly to the Doctor's recollection the very prominent situation which he fills; and at the same time to request him (as a great

---

* Other evidence than round assurance and mere ipse dixits will now be expected.

a great exemplar of the practice of vaccination) immediately and henceforward to discontinue the unwise and unwarrantable custom in the Central House of *breaking down pocks, at all stages, from their first formation till they have acquired their full dimensions, or that he will not continue to assure the parents of children who have been so treated, that these children are always secure*; and that he will also immediately advertise the public of the important and salutary reformation that has taken place there.

Dr. Jenner has no where authorised such *breakings down*, such *lacerations* of the vaccine vesicle; he has, I believe, admitted, that sometimes the *accidental rupture of the vesicle may destroy its preventive powers*; *and in an Address of the Royal Jennerian Society for the Extermination of the Small-pox*, which Dr. Walker himself did me the honour to transmit me some months ago, I find enumerated among the *probable causes* of occasional failures, the following: I quote verbatim,—" or by the *genuine vesicle having been destroyed at an early stage, and the regular progress of the disease thus interrupted.*"

Dr. Jenner and all his attentive disciples acknowledge and assure us, and indeed we all know, that the vaccine principle is determined by the subtlest and nicest laws. If a single herpetic blotch, or a few blotches of inveterate psora, on a part of the skin the most remote from that where the vaccine fluid has been inserted, will now and then influence and totally transform its action; if the use of a lancet, slightly rusty, in the insertion of this delicate principle will be followed by the same effect; if the process of sealing a letter containing it, or carrying it a little time in our pockets, (and Dr. Walker has insisted at great length on these circumstances) will either render it wholly effete, or capable only of producing an imperfect or spurious affection; if other such trifling causes, which might be adduced, have been known to effect similar deteriorations, will it be yielding to visionary impulses, or dreaming dreams, if we ask Dr. Walker, with all the soberness and seriousness which the nature and importance of the question demand, whether such *breakings down of the pocks, in all their stages, from their first formation till they have acquired their dimensions*, or, in other words, whether such demolitions of an animal process preparing and elaborating a principle and a fluid of these exquisite susceptibilities, can be always inflicted on it with impunity, punity, or without injuring, now and then, in some degree, such a product? If the vaccine vesicle, as Dr. Jenner has observed, be a congeries of cells insulated with respect to each other, must we not consider this constitution of it as a wise and beautiful œconomy of Nature for preserving its precious contents from casualty and exposure. This, undoubtedly, is the inference which Dr. Jenner draws and wishes should be drawn from it; but ample and effectual for its ends as we must conclude this bountiful contrivance generally to be, can we not as readily believe, that the *entire and repeated breakings down* of the vesicle in all its stages, &c. &c. of which we have so often been guilty, must in some instances have left not *a single cell* either *uninjured or unbroken, and consequently sometimes, in some measure, defeated it?* The resources of the human constitution, in the restoration and renovation of diseased parts, and parts which have been destroyed, often claim our highest admiration and gratitude; and if, in some cases, nothing less than new exertions of creative power can effect a complete reproduction of original organization, we find, in general, however, that all the great purposes of usefulness and convenience are obtained for us; yet, it must be allowed, that now and then the powers of the constitution are inadequate to this. This it is which, I presume, sometimes happens in the practice of vaccination. We often see children scratching and tearing with their nails the early vesicle, and see it reproduced again in most instances sufficiently perhaps, and place the subjects of it in the state of incomplete vaccination; but does even this always happen? Such, however, I believe, is the antivariolous power of the vaccine principle, as it is derived to us from the Jennerian Practice, under all the rude treatment, and under all the accidents to which the vesicle has hitherto been exposed, that the great purpose, for which we have so much abundant reason to conclude it has been given us, is probably *always obtained by it in constitutions of the lowest and even ordinary variolous susceptibility.* But when it shall happen that a degree of injury has been inflicted on the vaccine process in a subject of the higher or highest variolous susceptibility, annihilating or deranging it so much, that the slightest and most imperfect impression only of vaccinity can be transferred to the system by it; is it not very reasonable, is it at all injurious to the efficiency of the principle, to expect pect that regular and even numerous small-pox may, in some such instances, at some future period, occur? *

The conjecture respecting the *degrees of security* given to the constitution from the *imperfect action* of virus has

* Such a case, indeed, I have at this moment (under the observation of the medical men in this town, who, as usual, kindly and powerfully assist and co-operate with me) in the child of a very respectable officer in the Dock-Yard, Mr. Wallis, foreman of Shipwrights. This child, whom I vaccinated about three years ago, has now a numerous small-pox, but perfectly distinct, and unaccompanied with a shade of danger. At the end of the fourth day, they reached their full point of maturation, and on the fifth, all those in the face were shaded, and apparently beginning to dessicate. Vaccination took place in this child on one arm only, and Mr. Wallis assures me, *that I vaccinated children from this arm at different times.* On the cicatrix the usual indents, which are considered to be the vestiges of the respective cells to which we have alluded, are very indistinctly, if at all marked, and I think I see on it some seaminess; circumstances which shew, I think, that considerable violence had been inflicted on the vesicle. Mr. Wallis formerly suffered most severely from the small-pox; a stronger impression of bad small-pox than in his face is no where to be seen. This case, therefore, of small-pox may, *I flatter myself,* without violence to probability and truth, be *referred conjointly to high variolous* susceptibility and incomplete vaccination. This conclusion directly accords with many observations and facts which are in my possession, and which, truly, have not been made out and suborned to countenance and establish preconceived theories. Facts have here preceded theories. Although a case of this description of small-pox may, in a very few instances, be admitted to happen in the way of an exception to a general law of Nature, or, perhaps, referred to variolous insusceptibility at the period of vaccination; for it may be, perhaps, allowable to conjecture if we cannot yet believe, that where variolous susceptibility *has not yet existed,* that vaccination having here nothing to act on, may have left no anti-variolous impression on the System. To return to the vaccine cicatrix, I am inclined to think that this vestige, or sequela, has not yet been sufficiently attended to, as a test of vaccination; it will admit of a few remarks at least; neither is its extent, nor its depth, nor orbicularity, so decisively characteristic of vaccinity, perhaps, as those puncture-like indentations which we often observe on it. If the vesicle has been early and repeatedly opened by art, or has accidently sustained considerable friction, the ulcerative or purulent process which often succeeds, breaks down and destroys, it would seem, the insulation or independence of the respective cells, and leaves a cicatrix with nearly a smooth unbroken surface, or at most with very indistinct traces of indentations, and sometimes leaves it a little ridgy or seamy and uneven; neither, I believe, will these indents be seen in those cicatrices, however deep and extensive they may be, which follow those pustulous or spurious affections now and then occurring in the practice of vaccination. At present, therefore, I believe, that if these indentations are distinctly marked on a surface not less extensive than that of a split pea, we have a correct and satisfactory information of *complete vaccination.* Instantly and most gratefully, God knows, I shall relinquish these and any other ideas I have at any time advanced on this subject, the moment they are superseded by more obvious, more rational, and more probable conjecture and satisfactory explanation.

 been been, I believe, conceded me. I no where said, nor pretended to say, that no absorption or communication, or impregnation, or whatever it may be, takes place before the eighth day of vaccination. No man, indeed, is more convinced of the contrary fact; in pages 37 and 38 of a relation of some circumstances connected with vaccine inoculation which lately happened in this neighbourhood, and published by me about two months ago, I have adduced some arguments and facts to prove this; and stronger than these I can adduce: I will mention one which is well known; if we vaccinate a child to day in one arm, and the same child on the other arm four, five, or six days hence, the vesicle on the latter will, by the time that on the former is completely formed, have overtaken it. Now this cannot happen if the system is not under some vaccine influence at the time of the second inoculation. But I have said, and I repeat my assertion and belief at this time, that *all the effects* of the process of vaccination *derivable in and necessary for some constitutions, are not completed before nor at this stage* (the eighth day) *of the vesicle, where this has been previously and repeatedly ruptured, nor always where it has been preserved uninjured and unbroken to this period of the process only.*

Dr. Jenner's experiments, therefore, with caustic on the eighth day of the vesicle, in two subjects, are most unnecessarily, and without the least application, brought so triumphantly forward, for these only serve to prove what I have already steadily endeavoured to establish. But had Dr. Jenner, in conducting these experiments, chanced to have fallen on two subjects of high variolous susceptibility, and it may very poperly and fairly be added too, had the vesicles been daily broken down from their earliest formation, until the eighth day of their growth, (with respect to these latter circumstances, Dr. Jenner is silent in his account of the experiments) some secondary variolous affection, I have no doubt, would have taken place; and on this ground, I entertain as little doubt, that in a succession of two hundred such experiments at most, secondary and numerous variolæ would have occurred; in general, however, I believe the subjects of such experiments will enjoy many, if not all the advantages of complete vaccination, viz. complete protection in by far the greater number of instances, and exemption, probably, from danger in all.

When Dr. Walker thought it would serve him to glance at my late pamphlet, and tell your readers what they knew indeed indeed before, that in reviewing it you had observed, it contained several pages of theory and some of hypothesis, it certainly did not suit his views to remind them of what you further announced, at the same time, that the pamphlet contained *facts in which the whole world was concerned*; than which, I have always thought, a greater commendation of it could not have been given. To every man of the least reflection, it must unquestionably appear, that my faith in the practice of vaccination is not less than that of Dr. Walker, or any other vaccinist. But it will be seen also, that I have always endeavoured to rest my faith here on principles and foundations as physical and rational as possible; and if my humble and honest attempts to place this valuable discovery on such grounds shall prove entirely abortive, I have no doubt that my object and endeavours will be considered by some sober friends of vaccination at least as commendable, by none, assuredly, so visionary and terrifying, as Dr. Walker's imagination has drawn them. When I suppress or distort a fact, my hand, I hope, will cease to direct my pen.

As Dr. Walker is a plain man, no doubt loves truth, and, I heartily believe, means well to vaccination, he will receive the following information as a mark of respect for and attention to this practice, which is intended by it; and not as an incivility directed to himself, which is wholly disclaimed:—that at Plymouth we have for some time thought he has not, with respect to vaccination, put himself in the best posture of defence, nor selected the most effectual and approved weapons. When my avocations permit me, I shall not, I believe, decline with Dr. Walker a candid and generous discussion of any circumstances connected with vaccination, which may arise in course of our respective practices. As a preliminary arrangement, however, in favour of the Journal, and the patience of its readers, let us not hereafter talk a great deal about visions, and veins, and notions, and nonsense! but let us recollect that the times and circumstances are critical and imperious; and therefore, on the score also of science, of vaccination, and our own credit, demand every consistency in conduct that can possibly be observed. A fine example for our imitation presents itself in Dr. Jenner, of whose valuable friendship and luminous correspondence, with equal pride, happiness, and gratitude I mention it, my late papers have not for a moment deprived me.

RICHARD DUNNING.

*Plymouth Dock, May* 13, 1805.

